# Juggling on a rollercoaster? Gains, loss and uncertainties in IVF patients' accounts of volunteering for a U.K. ‘egg sharing for research’ scheme

**DOI:** 10.1016/j.socscimed.2013.03.002

**Published:** 2013-06

**Authors:** Erica Haimes

**Affiliations:** PEALS (Policy, Ethics and Life Sciences) Research Centre, School of Geography, Politics and Sociology, Newcastle University, Newcastle upon Tyne, NE1 7RU, UK

**Keywords:** UK, Eggs, IVF patients, Stem cell research, Mitochondrial

## Abstract

The past decade has seen a growth in demand for human eggs for stem cell related research and, more recently, for mitochondrial research. That demand has been accompanied by global debates over whether women should be encouraged, by offers of payments, in cash or kind, to provide eggs. Few of these debates have been informed by empirical evidence, let alone by the views of women themselves. This article addresses that gap in knowledge by presenting findings from a UK investigation, conducted 2008–2011, which is the first systematic study of women volunteering to provide eggs under such circumstances. This article focuses on the views and experiences of 25 IVF patients who volunteered for the Newcastle ‘egg sharing for research’ scheme (NESR), in exchange for reduced IVF fees. This was an interview based study, designed to gain understandings of volunteers' perspectives and reasoning. The interviews show that volunteers approached the scheme as a way of accessing more treatment in pursuit of their goal of having a baby, against a landscape of inadequate state provision of treatment and expensive private treatment. The process of deciding to volunteer raised a wide range of uncertainties about the consequent gains and losses, for women already in the uncertain world of the ‘IVF rollercoaster’. However, interviewees preferred to have the option of the NESR, than not, and they juggled the numerous uncertainties with skill and resilience. The article is as revealing of the ongoing challenges of the UK IVF bio-economy as it is of egg provision. This article adds to the growing body of knowledge of the contributions of tissue providers to the global bio-economy. It also contributes to several areas of wider sociological interest, including debates on the social management of ‘uncertainty’ and discussions at the interface of sociology and ethics.

## Highlights

► First study of IVF women volunteering eggs for research in return for compensation. ► Addresses debates on the ethics of encouraging women to provide eggs for research. ► Presents original data to address concerns about inducement and commodification. ► Contributes to worldwide debates about the uses of reproductive tissues in research.

## Introduction and background

In 2001 the UK became the first country to permit the use of human eggs in somatic cell nuclear transfer (SCNT or ‘therapeutic cloning’) research, followed by China, Japan, Singapore, South Korea (Mayor, 2004) then Australia, Sweden, Spain, Israel and India (Waldby, 2008:20); in the USA it continues mostly through private funding (Roxland, 2012). Research into mitochondrial disease has created further demand for human eggs (Human Fertilisation and Embryology Authority (HFEA), 2011; Nuffield Council on Bioethics (NCoB), 2012). Eggs are provided either by women undergoing in vitro fertilisation (IVF) or women in the general population; IVF patients can provide either ‘failed-to-fertilise’ eggs (which are no use for their treatment) or ‘fresh’ eggs collected before fertilisation has been attempted (whose potential to assist their treatment has therefore not been tested).

Numerous debates have arisen over the social and ethical challenges involved in acquiring eggs for research (Haimes, Taylor, & Turkmendag, 2012; Waldby & Carroll, 2012), highlighted by the ‘Hwang scandal’ which revealed falsified results and the likely abuse of women persuaded to provide eggs (Baylis, 2009). One contentious issue is whether women should be given any financial, or other, return, either for the eggs, or for undergoing the possible risks of ovarian stimulation and egg collection (Egli et al., 2011). The European Society of Human Reproduction and Embryology endorsed payments to egg providers, arguing that compensation to IVF providers should be more modest than for non-IVF providers as IVF patients undergo interventions as part of their treatment anyway (Pennings et al., 2007:1210). However, the idea of payments has attracted criticism around the world: are they undue inducements compromising women's autonomy and ability to give informed consent; are they exploitative of poorer women; are they payments for eggs, thereby contributing to the commodification of human body parts (Roxland, 2012)? Some feminists called for a moratorium on egg extraction for research (Plows, 2011:52–3), while others support payments to egg providers (Waldby, 2008). Roberts and Throsby (2008:160) suggest a moratorium fails ‘to address the complexity of women's relationship to reproductive technology and biomedicine’.

Few of these debates are informed by empirical evidence (Braun & Schultz, 2012), let alone by research that investigates the perspectives of women volunteering to provide eggs (Klitzman & Sauer, 2009). Our study addresses that gap in knowledge by investigating the views and experiences of IVF patients volunteering for the UK-based ‘Newcastle egg sharing for research’ scheme (NESR). In this scheme, ran by the Newcastle Fertility Centre (NFC) and established to provide eggs for nuclear transfer research, patients were given a reduction of £1500 in the fees for one IVF cycle when they agreed to provide 50% of their fresh eggs from that cycle. Full fees were approximately £3,000–£3700 per cycle (NFC, 2012). The NESR has a controversial history. It arose from collaboration between the NFC and the Northeast England Stem Cell Institute, whose scientists argued that better SCNT results would be achieved if they could use fresh eggs rather than potentially faulty, ‘failed-to-fertilise’ eggs (Stojkovic et al., 2005). The scheme initially received only a provisional licence from the HFEA, subject to the outcomes of a public consultation (HFEA, 2006). It received full approval in 2007, an ‘incoherent’ decision according to Braun and Schultz (2012:15) because it contradicted existing HFEA policies of non-payment for gametes and embryos; the HFEA responded that since they allowed ‘egg sharing for the treatment of other couples’ (EST), which gives heavily subsidized fees to the egg provider, ‘egg sharing for research’ (ESR) should also be allowed. Roberts and Throsby (2008) commented that NESR egg providers were being ‘paid to share’; the Singapore Bioethics Advisory Committee expressed ‘serious concerns’ that the NESR was exploitative (SBAC, 2008: 4.23).

However, the U.K. Medical Research Council (MRC) funded the nuclear transfer research (NT), including the discount to egg providers. They also funded our independent investigation of volunteers' experiences. Our central research question was ‘does egg sharing for NT research, in exchange for reduced IVF fees, entail social and ethical costs for those coming forward to participate in the scheme?’ This was a deliberately broad question, designed to maximise the opportunities to understand the scheme from the perspectives of the women themselves. It was thought that these perspectives would include, but also go beyond, views about payment. As the first empirical investigation of the perspectives of IVF patients volunteering to provide eggs under such terms, this study makes several contributions: it establishes the importance of understanding such schemes from the perspectives of women volunteering; it raises questions for egg acquisition schemes worldwide; it opens up ‘bioethics’ debates to social science investigation and analysis (Haimes & Williams, 2007); it adds to the growing body of knowledge of the contribution of tissue providers to the global bio-economy (Almeling, 2011; Waldby and Cooper, 2010).

At the time of our study the terms of the NESR were: (i) women had to volunteer as potential egg providers, in response to media coverage or clinic information leaflets; clinicians could not ask them directly for eggs; (ii) women should have had IVF previously, to confirm it was needed, and be aged 21–35; (iii) the consenting process should be conducted by an independent research nurse; (iv) if women produced six or more fresh eggs, they kept 50% and researchers received 50%, allocated one-by-one, on retrieval; if women produced an odd number of eggs they retained the extra one; if women produced five or fewer eggs they kept them all and still received the discount; women could choose their own, higher, threshold of eggs before the agreement was triggered; (v) women could change their minds up to egg retrieval but had to pay the full fees.

We have addressed the question of ‘exploitation’ and the NESR elsewhere (Haimes et al., 2012). In this paper I present a wider range of data, to provide contextualised ‘specificities’ (Roberts & Throsby, 2008:160) of how the NESR is experienced, and shaped, by women volunteers. This complements Roberts & Throsby's (2008:160) insightful, text-based, analysis of the ‘discursive construction’ of the NESR with the volunteers' lived experiences. The data presented here focus on how the IVF interviewees approached the decision to volunteer in the first place. They show that women situated their decision within a complex array of uncertainties about the gains and losses associated with giving up fresh eggs while receiving a discount, in the contexts of the already uncertain world of IVF (Ehrich, Williams, Farsides, & Scott, 2012) and of the UK IVF bio-economy. These insights help to answer Roberts and Throsby's (2008:160) question ‘as to why anyone undergoing fertility treatment would donate eggs potentially useful for their own treatment’ and illuminate the normative debates about payment, inducement and commodification in egg provision.

## Methodology

This was an interview based, inductive study, designed to gain understandings of the perspectives and reasoning of NESR volunteers. ‘Volunteer’ refers to women and couples who came forward as potential egg providers; ‘provision’ is used to avoid the more contestable discourses of ‘sharing’, ‘donating’ or ‘gifting’ (Almeling, 2011). Our project was approved by the local research ethics committee in 2008. The author is employed at the same university as that to which those running the NESR are affiliated; however, our project, though conducted with the full co-operation of the NFC, is completely independent. A Project Advisory Group (PAG) ensured analytical objectivity was maintained. Members included a senior scientist with a public record of opposing the NESR and SCNT research using human eggs and senior colleagues from Sociology, Law and Philosophy from other universities; they advised on study design, interviewee recruitment, data collection, analysis, and dissemination.

Fieldwork was conducted from 2008 to 2011. Since one of our goals was to understand whether *volunteering* created any social and ethical costs, we approached all women who had volunteered for the NESR. This included women who had gone on to provide eggs, women who had been accepted for the scheme but withdrew, and women who were not accepted, since we wanted to know the reasons for withdrawing and the effects of not being accepted. Volunteers were contacted by letter and information leaflet, sent on our behalf by the NFC, requesting participation in an interview. Those who agreed returned a signed consent form to us. Thus, we had no contact with patients unless, and until, they agreed to participate in our study and the NFC did not know which patients agreed to help us. Volunteers who had had recent IVF were contacted at least six weeks after their pregnancy test results. A total of 246 letters were sent, resulting in 25 interviews with IVF patients who had volunteered for the NESR. Seven of the 25 had been accepted for the NESR and had provided eggs; three were accepted but had withdrawn; 15 did not progress beyond the early stages, some of whom had been deemed unsuitable. While 25 in-depth interviews provided more than sufficient data to reach thematic saturation and to provide rich insights into the volunteering experience, further comparisons between the experiences of providing eggs, withdrawing, and being rejected, could have been made if more interviews within these sub-categories had been possible. Nonetheless, the data presented below focus on issues that had to be considered by *all* IVF volunteers, whether or not they provided eggs.

The author conducted all the interviews, enabling ongoing analysis and progressive focussing. The aide-memoir covered: demographic details; fertility history; experiences, and evaluations, of the NESR; views on broader debates on providing and acquiring eggs for research, and reflections on their volunteering experiences. However, the interviewees shaped the discussions, introducing topics and terminology relevant to their experiences. Most interviews lasted 60–90 min; all were fully transcribed. Transcripts were checked, anonymised and de-identified by the Research Assistant; the author and the RA then independently identified lists of major themes which were systematically compared and refined; initial coding of randomly selected transcripts led to further refinement of major themes; an agreed list of 19 themes was then used by the author to code all the transcripts. Constant comparison and category-building procedures, followed by category mapping (Silverman, 2001) were used to sort data extracts. These analytical strategies and procedures were discussed with the PAG. As part of our wider study, we also conducted 42 interviews with other groups for a range of comparative purposes but I focus here on the accounts of the 25 IVF volunteers.

## Volunteers' views and experiences

I shall outline interviewees' views and experiences of volunteering for the NESR under three headings: (1) their experiences of fertility problems and treatment; (2) the issues they considered when deciding to volunteer; (3) their overall evaluations of the NESR.

### The infertility and IVF rollercoaster

All interviewees echoed, throughout their interviews, the now well-documented (Spar, 2006:1–30) difficulties of infertility and of IVF treatment (Aarts et al., 2012). Given that these stresses are widely acknowledged I shall keep this material brief, in order to retain space for newer insights from our study. Nevertheless, it is important to hear some examples of interviewees' graphic descriptions. One described the *‘mental toll’* of infertility; ‘*it really impacted my life badly’* (M06:1020–68 and 860–914). Another said, *‘it's a rollercoaster every month’* (M25:6806). Interviewees described IVF as *‘horrendous, physically, emotionally, financially’* (M11:980–1012) and as *‘a hard experience, very, very emotional*… *a real rollercoaster’* (M19:1279–91). The first cycle of IVF was the most difficult; at least in later cycles, ‘*I knew what to expect’* (M24:428–528). IVF success rates remain low: the 2009 HFEA figures (the most recent available) show that for women aged under 35 years, 32.3% of IVF treatments using fresh eggs resulted in a live birth (HFEA, 2012a). Nonetheless, these interviewees were seeking further treatment. In light of the descriptions above this suggests that IVF itself can be a combination of loss, gain and uncertainty: it is horrendous to go through, but if it results in a baby, it is probably worth it (Thompson, 2005). It is this desire for a baby that emerged as the volunteers' central, sense-making, frame, as they explained their approach to the NESR.

### Losing eggs, gaining the discount, juggling uncertainties

In deciding whether to volunteer, interviewees had to weigh the prospect of reducing their fresh eggs by 50% against the value of the discount. Central to their accounts was uncertainty about what they were gaining and losing especially as their handling of these two elements was embedded within a range of other, entangled, uncertainties.

#### Losing eggs?

Since fresh eggs (in contrast to failed-to-fertilise eggs) ‘are always potentially useful to patients’ (Roberts & Throsby, 2008:160; Waldby & Carroll, 2012) and since patients regard producing sufficient eggs as an early successful step in IVF (Haimes & Taylor, 2009), it is unsurprising that, for most interviewees, contemplating losing 50% of those eggs was a significant consideration. Most interviewees (including all seven who went on to provide eggs) reported having produced ‘lots’ of eggs in previous cycles, ranging from 17 to 46:

*‘*… *it's not something that somebody who wasn't producing a lot*… *could really think about, because it's halving their chances’* (M02:469–510).

Previous experience also alerted interviewees to the variability in these numbers: one woman who had produced ‘lots’ previously, only had three in her most recent cycle: *‘Now I know that if I was to do it again I probably wouldn't have as many as I thought’* (M09:772–85).

Interviewees also knew from previous IVF the complex relationship between numbers of eggs, numbers of viable embryos and the likelihood of a baby (termed elsewhere ‘the calculus of conception’ (Haimes & Taylor, 2009:2143)). One said, ‘*we tried to do it statistically’*, using numbers from a previous cycle to decide the minimum number of eggs to keep before triggering the NESR arrangement (M10:372–409). Another calculated:

*‘*… *the more eggs you have, the more chance they give us, so [giving some up] was a big worry. So even though we had a lot of eggs, 19,*…*maybe only 10 [fertilised]*…*I'd half my chances again if we [only] had half the eggs I'd made’* (M11:687–702)

Another said, ***‘****you never know*… *anything under [ten] I was keen to keep*… *You always think on the day “oh how many are we going to have? How many are going to fertilise?”’* (M05:582–657).

Contemplating reducing their eggs by 50% was made easier by the terms of the NESR: *‘[It] was reassuring to know that if you did have a small amount you weren't going to lose half’* (M03:551–98). They could also withdraw but at a cost: ‘*You can always change your mind if you're bordering on seven, eight and you don't want to risk it. But you do have to pay the full amount!’* (M07:394–442).

Interviewees expressed little interest in changing the policy on the number of eggs a woman should have before the agreement was triggered; they simply wanted ‘enough’ to feel confident that providing 50% to research would not damage their chances of pregnancy. Therefore, from the outset of considering volunteering, women had to work with uncertainties about the number of eggs they would have and the effect of reducing that number by 50%. They combined previous IVF experiences with the terms of the NESR to try to judge the risks of volunteering.

#### Gaining the discount?

Overwhelmingly, interviewees' perspectives on the reduced fees were tied to concerns about (a) the costs of private treatment and (b) levels of state-funded IVF in the UK. Once the possibility of IVF had arisen, interviewees had immediately started thinking about how to manage the potential costs. They were confronted by the entanglement of treatment, babies and money long before they heard about the NESR: *‘money is the thing with IVF, unfortunately, that's what it boils down to’* (M21:344–57).

**(a) Private fees:** The costs of private treatment were raised by almost all interviewees: ‘*a hell of a lot’* even half price (M16: 943–62); ‘*really, really expensive’* (M28:255–311). One couple suggested clinics have ‘*captive audiences’* so can charge what they want (M01:957). There was worrying uncertainty about how high fees would eventually be since, when they embark on treatment, patients cannot be sure how many procedures, drugs or cycles they will need. For most interviewees, the NESR offered the chance of a cycle that otherwise would not have been affordable or reassurance that they would be able to afford treatment more easily. The discount put a single cycle ‘*within our reach’* (M15:857–909); *‘we would not have been able to do another cycle then if we didn't have the assistance of the egg sharing scheme*…*’* (M03:600–56). One woman said she could ‘afford’ more treatment by using credit cards: *‘I would just have been more in debt [laughing] and I wouldn't have probably been able to have it as frequently’* (M07:557–618). Another said, *‘*…*financially [IVF] nearly ruined us the first time*… *this really would have been probably our last go, financially and emotionally. But we didn't have the money for a full treatment*…*’ (*M06:943–77).

Interestingly, there was little relationship between interviewees' sense of the affordability of any/further IVF and their incomes. Interviewees reported annual household incomes between £15,000–£70,000 (average of £45,468); the average household income for north east England, where the NESR is based, was £28,600 in 2008 (Office for National Statistics, 2011). The seven interviewees who provided eggs were among the higher earners, with an average household income of £50,786. Higher earning interviewees were no more convinced they could afford private IVF because of the uncertainties about the eventual costs.

**(b) State funding:** The limited availability of National Health Service (NHS)-funded IVF was raised, unprompted, by every interviewee. They were particularly troubled that NHS funding is usually not available if one partner already has a child:

*‘From an NHS point of view I would have had to go through life without having somebody really close to me, being little, and seeing them grow up, because [partner] had two grown up children’* (M24:1169–1205).

Some argued for greater flexibility in accessing NHS funding, suggesting a reciprocal arrangement: *‘If I was getting NHS help they could have had my eggs for nothing’* (M20:114–23). One woman angrily said that people who do not approve of the NESR should,

*‘set up some scheme where people can get funding, other than having to wait on the NHS*… *then you can have some of my eggs for research, but if you won't give me the free treatment then at least give me reduced fees so you can have the eggs'* (M03:1598–1646).

The positioning of NHS-funded cycles in relation to the NESR was clear: ‘I would prefer the NHS waiting list was not so long [as] that would be my first option and I always wanted [the NESR] to be a last option’ (M17:937–71). This was echoed by others whose preference would be to have NHS treatment and not have to consider giving up eggs.

U.K. national guidelines recommend that IVF patients who meet local criteria (which vary but often have stipulations about age, marital status, pre-existing children) should be allowed three cycles of NHS-funded IVF (National Institute of Clinical Excellence, 2004). A recent study shows that that level of provision would place the UK amongst the lowest providers of state-funded IVF in Europe (Photopoulos, 2012); approximately 75% of health regions in England and Wales do not provide even this (All Party Parliamentary Group on Infertility, 2011). Extensive lobbying in the north east of England, including by the NFC, led to the five local NHS funders implementing the national guidelines. This might have reduced the number of egg providers for the NESR; their target was 80, but they recruited 42 (Choudhary et al., 2012).

**(c) ‘Money isn't everything’:** Given the cost of private treatment and the anticipated difficulties accessing NHS-funded treatment, the NESR discount was clearly important. However, interviewees were cautious in allowing it to determine their actions. It was: *‘not the be all and end all, but*… *it is a consideration’* (M19:768–95). ‘*Money isn't everything, success is everything*… *whilst you’ve got to take finances into account you*… *don't want to limit any chance of success'* (M01:776–92). A couple who withdrew said,

*‘*…*we started out thinking “financially this is a great idea”, morally we agreed with it* …*[but] when we didn't have that many eggs*… *we felt, “we don't care about the money, we should keep all of these eggs*…*”’* (M10:863–96).

Significantly, although ‘egg sharing for treatment’ (EST) for other couples would have given them almost free treatment, all but two interviewees rejected this:

*‘ it would be too hard*… *to even contemplate that somebody else was having a child from an egg that I had produced, if I was never going to be able to*… *But the egg sharing for research, there weren't any doubts about that’* (M03:346–410).

Interviewees judged the discount as a proportion of the total cost of private treatment and therefore in terms of the extra cycles they would be able to afford:

*‘If they said, “Well, you can have it for 25%”, then that's four chances and that's the way we were looking at it, rather than £3000 or £1500*…*’*(M10:1418–61)

In discussions about whether they would give eggs for no return at all, interviewees said they would, if: there were eggs that were no good for their treatment; there were eggs that they (rather than anyone else: Scott, Williams, Ehrich, & Farsides, 2012) deemed ‘surplus’ to their needs; they had had a baby; they had decided to end treatment. Until such circumstances arose they could not give them for free as, *‘I've still got that tiny bit of hope so I need whatever's there!’* (M21:1031–48).

The data so far suggest that volunteering for the NESR occurs in the absence of interviewees' preferred options of having greater access to NHS-funded treatment or being able to afford private fees, and therefore, in both cases, of being able to retain all their eggs. Interviewees approached the NESR as a scheme through which they could access cheaper treatments, as a consequence of which they would give half their eggs to research, rather than as a scheme persuading them to give up their eggs (even though they were clear that it existed to acquire eggs for research). This is an important distinction, as it indicates that volunteers approached the NESR with their own goals, rather than merely responding to external inducements. This is further evidenced by the view that cheaper treatment is not the ‘be all and end all’; having a baby is what really matters. Nonetheless, the discussion about giving eggs ‘for free’ reveals the circumstances in which they would *prefer* to provide eggs.

#### Juggling further uncertainties

While reduced fees and the loss of fresh eggs are the two aspects of the NESR that have attracted most debate, for interviewees there was a complex array of other biographical, emotional and practical considerations that also had to be taken into account, and sometimes offset against each other, in making the decision to volunteer. Each consideration was rife with uncertainty; together they constituted the entangled backdrop to interviewees' approaches to the NESR.

These wider uncertainties included: the benefits of accessing more treatment against the additional stresses of enduring more cycles; whether participating in the NESR might result in fewer embryos to freeze; comparing the chances of success from fresh embryos from extra treatment to the chances of success from frozen embryos that they might have had; whether, particularly for older women, the NESR would mean they could have more treatment, more quickly, than spending precious, ageing, time saving up; the fear that the NESR might not last for long so they would have lost an opportunity for cheaper treatment; whether it was better to volunteer for the NESR or go to other clinics which might claim better success rates.

Skill and resilience were deployed in managing these multiple uncertainties as interviewees were pulled in different directions at once:

*‘I'm not normally a gambling person but you've got to take that risk*… *you start with nothing; hopefully you'll end up with something but you might end up with nothing*… *but you could have a whole batch of eggs that are all a bad batch [anyway]*… *So I think you would take that chance [with the NESR]*… *and if your first [cycle] doesn't work at least you've got that second attempt rather than sitting*… *in the depths of depression worrying about where you're going to get your next £2000–3000 from’* (M28:1060–1152).

This interviewee later said, *‘It is [complicated] when you start thinking about it*… *[that's] probably why I only think about it in bits!’* (M28:1657–94). However, it was not easy for volunteers to separate out the different elements of ‘IVF + NESR’, because of all the unknowns:

*‘She said “if you're producing this many [eggs] there should be no reason why you wouldn't do [so] next time”, but you never know. And*…*however many [embryos] you freeze, they're less successful for implantation*… *so we thought, “rather than paying to have them frozen, we may as well go through the next treatment half price!”(laughs)… you have to laugh about it because you'd cry if you didn't*…*I think [the NESR] was only [available] for a year but because I would have still been under 35, if the NHS one hadn't come up, we probably would have done [NESR] again because I would have still fulfilled the criteria with my age*…*’* (M05:664–711).

Another said: *‘It's lots of things going on*…*’* (M10:782–856). This constant juggling of unknowns led some to reconsider participation. Two interviewees who withdrew said of the NESR: *‘it's not two for the price of one*… *because it's double the emotional cost’* (M10:1544–62); and, ‘*The [NESR] did alleviate some financial pressure of not having to find the full three thousand pounds but it doesn't take away the emotional pressure, the physical pressure’ (M11:1014–44).*

For others though:

*‘I think [the NESR] would be a last option. I would still worry that it didn't give us as good a chance*… *But if it was an option where it was to do with time and obviously financial, then it's a last hope really.’* (M23:688–733).

Therefore, a representation of the NESR as a relatively simple, linear, relationship between the paucity of state funding, the need for private IVF, the offer of reduced fees and the consequent decision to provide eggs (Braun & Schultz, 2012:9) is not an adequate depiction of volunteers' experiences. Rather, interviewees juggled numerous uncertain considerations, which all had to be judged simultaneously, *in relation to each other*. Since it is never clear just which factor will be the key to success, all the ‘balls’ have to be kept in contention. Given that interviewees likened IVF to a rollercoaster, ‘IVF + NESR’ can be likened to trying to juggle on a rollercoaster.

### Did the gains outweigh the losses? Interviewees' evaluations of the NESR

Deciding to volunteer for the NESR involved a complex navigation through multiple uncertainties. Having decided to volunteer, interviewees had mixed experiences: 15 did not progress beyond the initial stages of completing a questionnaire to determine suitability while ten others were accepted; three then withdrew and seven went on to provide eggs. There is insufficient space to detail those subsequent experiences here, but it is useful to present a brief indication of how interviewees regarded the NESR, at the time of interview.

Overwhelmingly, interviewees' assessment of volunteering for the NESR, whatever their eventual pathway, was, *‘I would do it again, definitely. No regrets at all’* (M06:1488–98). As detailed elsewhere (Haimes et al., 2012) they liked that: they had to initiate participation rather than be asked by clinicians; there was no direct or indirect pressure to participate; they had time to consider their decision; they felt well informed; they were gaining the chance of more treatment; they could change their minds up to egg collection; the NESR was part of IVF treatment so nothing extra was involved, and they were helping research.

All those who provided eggs endorsed these evaluations. Of the three interviewees who withdrew, one did so because she had the opportunity of an NHS-funded cycle which was successful; she was considering volunteering again, since she was not eligible for NHS funding now she had a baby. Another conceived naturally while waiting for her NESR cycle. The third changed her mind when she did not have as many eggs as she had hoped. The decision to withdraw, amongst this cohort at least, did not arise out of complaints about the scheme. In fact, two of these women said, *‘now we know more what it's about and how we're going to feel, we're going to try egg sharing again and see it through this time*…*’* (M10:1165–79) and *‘I think it [NESR] was a great opportunity*… *to have that choice was fantastic’* (M11:1300–1318). A frequent response from those who did not progress beyond the early stages was that they had no regrets volunteering. When asked whether the NESR raised the hopes of patients, only to dash them when they were not accepted, one replied: *‘Any hope is better than no hope’* (M15:1708–65), a view echoed by others: *‘the whole process [of IVF] is disappointments and getting your hopes up*… *I would ask them again, if I could (laughing)! Take any chance you can get!’* (M21:1384–1402).

Thus the NESR received overwhelming endorsements, even from those who withdrew or were unable to participate. It could be argued that the NESR takes advantage of the patients' financial difficulties but this was not a view expressed by the interviewees themselves. Rather, they regarded the very existence of the NESR as a gain. While ‘IVF + NESR’ brings its own challenges, it is, nonetheless, ‘IVF+’, it is another option.

## Discussion

Recently, the HFEA approved compensation of up to £750, for expenses and loss of earnings, to anyone providing gametes for treatment or research (HFEA, 2012b) and the Nuffield Council on Bioethics recommended a pilot scheme be conducted to assess the effects of offering financial compensation to non-IVF egg providers (NCoB, 2011:209–210). How best to organise the provision of gametes for research, including for the expanding field of mitochondrial research (NCoB, 2012), is clearly still open to debate.

This study shows the importance to these debates of hearing from those volunteering to provide eggs. The IVF interviewees frame volunteering for the NESR in terms of the challenge of how to have a baby and therefore how to access sufficient treatment to facilitate this. In partial answer to our central research question, these interviews suggest that volunteering for the NESR does entail some costs since providing fresh eggs for research is not an easy solution to the problem of accessing treatment. IVF is a rollercoaster, with sudden emotional, financial, physical highs and lows; the NESR then involves trying to juggle a wide range of other uncertainties of possible gains and losses while on that rollercoaster. It has been argued that IVF patients should not be confronted by these quandaries ‘at a time of particular vulnerability’ (Waldby & Carroll, 2012:525). That view would derive some support from the literature questioning the acquisition for research of fresh embryos from IVF patients (Ehrich, Williams, & Farsides, 2010; Haimes & Taylor, 2011; Scott et al., 2012), even if one acknowledges the crucial, contextualised, differences between eggs and embryos. However, these interviewees preferred to have the option of the NESR, than not, and, rather than buckle under these challenges, they decide to volunteer (having already, it should be noted, decided to continue with IVF). Through that decision they appear to gain both hope and *some* control over their circumstances. Given the not uncommon portrayal of IVF patients as desperate victims of their infertility, it is important to recognise that uncertainty can be managed with skill, patience, ‘creativity and critical agency’ (McLaughlin & Goodley, 2008:331).

While it is important not to overstate the degree of control that any IVF patients have over their circumstances (Spar, 2006) it is nonetheless useful to understand how interviewees managed these uncertainties. Gross (2012:434) reminds us of Simmel's suggestion that uncertainty (or ‘nonknowledge’) is a feature of the ‘technicized society’; it becomes incorporated into everyday life, where ‘the right strategy cannot be to do nothing or to wait until certain knowledge is available’. Gross argues that it is the acknowledgement of the role that ‘nonknowledge’ plays in everyday life, rather than complex analyses of ‘risk’, that leads to a better understanding of everyday actions. Few interviewees (who are very experienced at living with uncertainty) engaged in detailed calculations of risk; most focused instead on their hope (itself a form of uncertainty: Eliot & Olver, 2007) that extra treatment will increase the chances of pregnancy. Some reassurance that that hope is not misplaced comes from recent data from the NFC, not available at the time of interviews, indicating that the ‘live birth rate per treatment started’, for NESR egg providers, was 37.25% and 29.4% for matched comparators (Choudhary et al., 2012). However, the analogy of trying to juggle on a rollercoaster supports Roberts and Throsby's (2008) questioning of the claim that the NESR is a ‘win–win’ for both volunteers and researchers, exposing this as a gloss on volunteers' actual experiences.

Reference to interviewees' skills and resilience is not intended to underplay the fact that interviewees did not volunteer in circumstances of their choosing, when already in the position of trying for a baby in circumstances not of their choosing. Volunteering occurs in a context where private IVF fees are too high to manage easily, or at all, and where there is insufficient NHS-funded treatment; this supports Braun and Schultz's (2012:9) assertion that the attractiveness of ESR schemes will be higher where there is less state funding for IVF. It also lends weight to those querying high private fees in the UK and the inadequate implementation of national guidelines for NHS-funded IVF (Winston, 2011). If both these were improved, patients might then be in a position to provide eggs to research under less ambivalent (Brown, 2012:13) circumstances, in which the role of ‘choice’ becomes clearer. In the meantime, and in (an admittedly partial) response to the normative question of whether payments should be offered to women providing eggs, it is important to reiterate (Haimes et al., 2012) that the interviewees' positive endorsements of the NESR are clearly related to the specific socio-economic landscape of UK IVF provision and should not therefore be taken as a simple mandate to extend IVF ‘egg sharing’ schemes worldwide. Nor should volunteers' endorsements be used to justify offering payments to encourage egg provision from ‘non-IVF’ women, who occupy a completely different social position in relation to possible gains, losses and uncertainties; their perspectives require their own detailed investigations.

Our study also reveals the ‘thin’ understandings in the bioethics literature of how potential egg providers manage the concerns raised in that literature. By attending instead to the Geertzian ‘thick’ descriptions revealed by the inter-subjective particulars of interviewees' everyday lives (Haimes & Williams, 2007), it is possible to see how those bioethical issues are shaped by the IVF context. For example, on the question of informed consent, we have seen that interviewees invoked their previous experience of IVF (being one of the few certainties in IVF) to explain to themselves what it was they were volunteering to do. One could argue that their abilities to act autonomously, and to give better informed consent, would be compromised less by the offer of reduced fees and more by the lack of previous treatment (Carroll & Waldby, 2012; Haimes & Taylor, 2009). As Plows (2011:51) argues, we need to consider the ‘broader political and social background against which informed consent in a specific context is given by a specific woman’. Our study provides insights into both the broader background and the specific context of the NESR; further in depth studies of egg acquisition practices elsewhere need to be conducted to understand how those backgrounds and contexts shape volunteers' autonomy.

While the discount was clearly important in the decision to volunteer for the NESR, volunteers were cautious about providing eggs if they felt it would seriously compromise their chances of pregnancy. Also most did not volunteer for EST, even though that would give them almost free treatment. Therefore, reduced fees appear not to act as *undue* inducements persuading volunteers to act against their own interests (Hyun, 2006:630). This supports (Sandel's 2012:91) assessment that financial incentives are not inherently degrading of core values but vary ‘from case to case’, as does the ‘moral importance of the attitudes and norms that money may erode’. The interest in benefitting from reduced fees also suggests that NESR volunteers are not succumbing to appeals to ‘gendered altruism’ (Plows, 2011:51) and thinking they should ‘gift’ their eggs. Rather than asking whether potential egg providers are motivated by ‘compensation’ or ‘altruism’ (Egli et al., 2011), our research suggests these terms are not mutually exclusive. Interviewees would not ‘give their eggs away’ while pursuing their goal of a baby, but expressed strong values of future altruism for when that goal had been achieved or abandoned. Volunteers are not altruistic, or non-altruistic, by ‘nature’; rather, as the NCoB recognised, their actions are shaped by specific biographical, social and economic contexts (NCoB, 2011:140).

Discussions about payments tend to lead to discussions about commodification. This study suggests that, rather than being ‘paid to share’ (Roberts & Throsby, 2008) interviewees are *exchanging* eggs for treatment; the NESR might more appropriately be called an ‘egg exchange’ scheme (Haimes et al., 2012). Volunteers are potentially exploiting *both* the ‘use value’ and the ‘exchange value’ of their eggs (Brown, 2012:2–3); since they cannot access IVF on their preferred terms they can at least use their eggs to get the treatment they want. While this lends weight to the view that the NESR encourages the ‘commodification’ of human eggs (a contested term: Almeling (2011:3–21)) it could also be argued that such concerns hold less sway in the IVF context. IVF requires a fragmentation of bodies and the ‘entification’ of body parts which ‘become detached…from human bodies… [and] reappear as…usable entities…that are disposable and available for choice’ (Lie, 2012:1–5). IVF involves calculations of how different bodily fragments (e.g. sperm, follicles, eggs, embryos) contribute to a successful outcome (witness Choudhary et al., 2012). As we have seen from the interviews, IVF also involves calculations (partly based on the presence and quantities of those fragments) of the necessity for, and affordability of, further cycles. That is, routine IVF entails an entanglement of treatment, money, body parts and babies, but is rarely regarded as commodifying the human body. The interviews indicate ways in which NESR volunteers can benefit from these dual processes of entification and exchange, rather than be subjected to them. Equally, the interviews indicate that entification and commodification do not necessarily imply a devaluing of body parts. Eggs are both important and exchangeable; the one does not preclude the other.

Therefore, this study is a resource for the further exploration of the intertwining of the ‘descriptive’ and the ‘normative’, the moral and the social. Drawing on the work of Kant, Weber, Wittgenstein, Louch and others, we have argued elsewhere (Haimes & Williams, 2007:470–472) that these entanglements provide opportunities for collaborations between sociology and bioethics. Developments in bioethics over the last ten years suggest a similar interest in these opportunities (Molewijk and Frith, 2009).

This study also adds to the growing body of social science analyses (many cited here) of the social practices of provision, acquisition and uses of human reproductive tissue in research. Given the focus in this paper on presenting new data from the perspectives of women directly involved, there is insufficient space to detail the many wider connections that can also be made between this study and those of others in the global markets for other human tissues. Nonetheless, one very clear connection is the usefulness of an analysis of tissue providers' perspectives which enhance understandings of the ways in which they contribute to the global bio-economy, not least by turning attention towards the labourer, and her labour, rather than simply focussing on the products of her labour (Brown, 2012: 13). Such an approach can be usefully combined with those in other fields, such as Almeling's (2011) elegant analysis of the markets for, and ‘donors’ experiences of providing, gametes for treatment, to enlighten the ways in which situated specificities combine with socio-economic and ethical structures to facilitate, regulate or inhibit such tissue transactions.

Our study has shown that the NESR was welcomed, and managed, by IVF patients because of its position within the UK IVF bio-economy. It tells us as much about IVF, and its promises and problems, as it does about the uncertainties of volunteering for egg provision. In so doing, it alerts us to avoid treating reproductive transactions in social and bioethical isolation and instead to trace the threads of those transactions across the many different social domains in which they are embedded.
